# The Abundance Paradox of S100A8/A9 in Neutrophils: Functional Logic of Calprotectin Dominance in the Cytosolic Proteome

**DOI:** 10.3390/ijms27093889

**Published:** 2026-04-27

**Authors:** Kyung-Hee Kim, Byong Chul Yoo

**Affiliations:** 1 Department of Applied Chemistry, School of Science and Technology, Kookmin University, Seoul 02707, Republic of Korea; kyungheekim@kookmin.ac.kr; 2 Antibody Research Institute, Kookmin University, Seoul 02707, Republic of Korea; 3 Diagnostic Research Team, InnoBation Bio R&D Center, Seoul 03929, Republic of Korea

**Keywords:** calprotectin, S100A8, S100A9, neutrophils, granulopoiesis, nutritional immunity, innate immunity, tumor microenvironment

## Abstract

Neutrophils are the most abundant circulating leukocytes and are characterized by a proteome in which granule-associated proteins synthesized during granulopoiesis constitute a major fraction of total cellular protein, reflecting their preloaded effector nature in innate immune defense. A striking feature of neutrophil biology is the unusual abundance of the calcium-binding proteins S100A8 and S100A9, which together form the heterodimeric complex known as calprotectin. Early biochemical studies estimated that S100A8/A9 constitutes a substantial fraction of the soluble cytosolic proteome in neutrophils, with later studies often describing it as one of the most abundant protein complexes in these cells. Despite extensive studies on the antimicrobial and inflammatory activities of calprotectin, the biological rationale for this unusual abundance remains incompletely understood. In this review, we examine the structural, biochemical, and regulatory features of S100A8/A9 and explore the potential explanations for its high abundance in the neutrophil cytosol. We first discuss the unique organization of the neutrophil proteome and the transcriptional programs governing granulopoiesis that lead to large-scale production of neutrophil effector proteins. We then review the structural and biochemical properties of S100A8/A9, including its calcium-dependent conformational dynamics and high-affinity transition metal binding, which contribute to antimicrobial defense through nutritional immunity. Several functional hypotheses are considered to explain calprotectin abundance, including roles as an antimicrobial reservoir, a metal-sequestering molecule, a regulator of oxidative stress, and a source of damage-associated molecular patterns. Finally, we discuss the evolutionary logic of neutrophil protein preloading and the implications of calprotectin biology in inflammatory diseases and the tumor microenvironment. Resolving the abundance paradox of S100A8/A9 may reveal fundamental principles governing the organization of innate immune cell proteomes and provide new insights into the strategies used by neutrophils to achieve rapid and effective host defense.

## 1. Introduction

Neutrophils are preloaded effector cells in which granule-associated proteins synthesized during granulopoiesis constitute a major fraction of total cellular protein. They are the most abundant leukocytes in human circulation and represent a central component of the innate immune system. These short-lived granulocytes are specialized for rapid antimicrobial defense and are equipped with a preassembled arsenal of effector molecules that can be deployed immediately upon infection or tissue injury [1,2,3,4,5,6]. Unlike many other immune cells that rely on inducible transcriptional programs following activation, neutrophils function primarily as preloaded effector cells, storing large quantities of antimicrobial proteins and inflammatory mediators synthesized during granulopoiesis in the bone marrow [7,8,9,10,11]. This strategy enables neutrophils to respond within minutes to invading pathogens and inflammatory stimuli, even though mature neutrophils exhibit relatively limited transcriptional activity and a short lifespan in circulation [12,13,14,15].

In addition to their classical antimicrobial functions, neutrophils have emerged as versatile regulators of immune responses that interact extensively with both innate and adaptive immune pathways [5,8,16]. Accumulating evidence further indicates that neutrophils are not a uniform cell population but instead display substantial functional heterogeneity, giving rise to distinct subsets that participate in host defense, immune regulation, and tissue remodeling [17,18]. These findings have expanded the traditional view of neutrophils as simple antimicrobial effector cells and instead highlight their complex roles in inflammatory diseases and immune homeostasis [19].

Among the numerous proteins present in neutrophils, a striking and unusual feature of the neutrophil cytosolic proteome is the exceptional abundance of the calcium-binding proteins S100A8 (S100 calcium-binding protein A8) and S100A9 (S100 calcium-binding protein A9). These two proteins form a heterodimeric complex commonly known as calprotectin. Early biochemical studies based on quantitative analyses of neutrophil cytosolic proteins indicate that S100A8/A9 constitutes a substantial fraction of the soluble cytosolic proteome in human neutrophils, generally reported in the range of 30–45% [20,21]. These studies provide primary experimental evidence supporting the unusually high intracellular abundance of S100A8/A9. However, as estimates may vary depending on experimental conditions and analytical approaches, the available evidence is best interpreted as indicating a markedly enriched but variable proportion of S100A8/A9 within the neutrophil cytosolic proteome.

Therefore, rather than relying on a single absolute value, the available evidence supports a robust and reproducible pattern of disproportionate enrichment of S100A8/A9 within the neutrophil cytosolic proteome, underscoring its marked enrichment compared with typical signaling or regulatory proteins in immune cells. This interpretation is supported by proteomic and transcriptomic analyses of neutrophils, which collectively indicate a highly skewed molecular composition dominated by a limited number of highly expressed proteins [22,23].

S100A8 and S100A9 belong to the family of EF-hand calcium-binding proteins and are predominantly expressed in myeloid cells, particularly neutrophils and inflammatory monocytes [24,25,26]. The S100A8 and S100A9 subunits are encoded by adjacent genes within the S100 gene cluster on chromosome 1q21 and assemble primarily as non-covalent heterodimers in the presence of calcium [27,28]. This complex can further oligomerize into higher-order structures under physiological conditions and has been implicated in a wide range of intracellular and extracellular immune functions. Indeed, S100A8/A9 proteins have been extensively studied as mediators of inflammatory signaling, antimicrobial defense, and leukocyte recruitment, and they function as damage-associated molecular pattern (DAMP) molecules capable of activating innate immune receptors such as Toll-like receptor-4 and the receptor for advanced glycation end products [29,30,31].

A particularly well-established function of calprotectin is its role in antimicrobial host defense through sequestration of transition metals. By chelating essential divalent cations such as zinc and manganese, calprotectin can restrict microbial access to nutrients required for bacterial growth, a process known as nutritional immunity [32,33,34,35]. Through this mechanism, neutrophil-derived calprotectin contributes to the inhibition of microbial proliferation during infection and inflammation. In addition to antimicrobial activity, S100A8/A9 proteins have been reported to participate in diverse biological processes including regulation of leukocyte migration, modulation of reactive oxygen species production, and amplification of inflammatory signaling pathways [21,36,37].

Despite extensive studies on the biological activities of S100A8 and S100A9, a fundamental question remains largely unexplored: why do neutrophils devote such an unusually large fraction of their cytosolic proteome to this protein complex? Unlike structural proteins or metabolic enzymes that typically dominate cellular proteomes, S100A8/A9 are multifunctional inflammatory effectors, making their exceptional abundance a striking proteomic paradox in innate immunity. This disproportionate representation raises important questions regarding the functional logic and evolutionary rationale underlying calprotectin predominance in neutrophils. Several non-mutually exclusive hypotheses have been proposed to explain this phenomenon, including antimicrobial reservoir functions, metal sequestration during nutritional immunity, inflammatory signaling, and intracellular stress regulation (Table 1).

**Figure 1:**
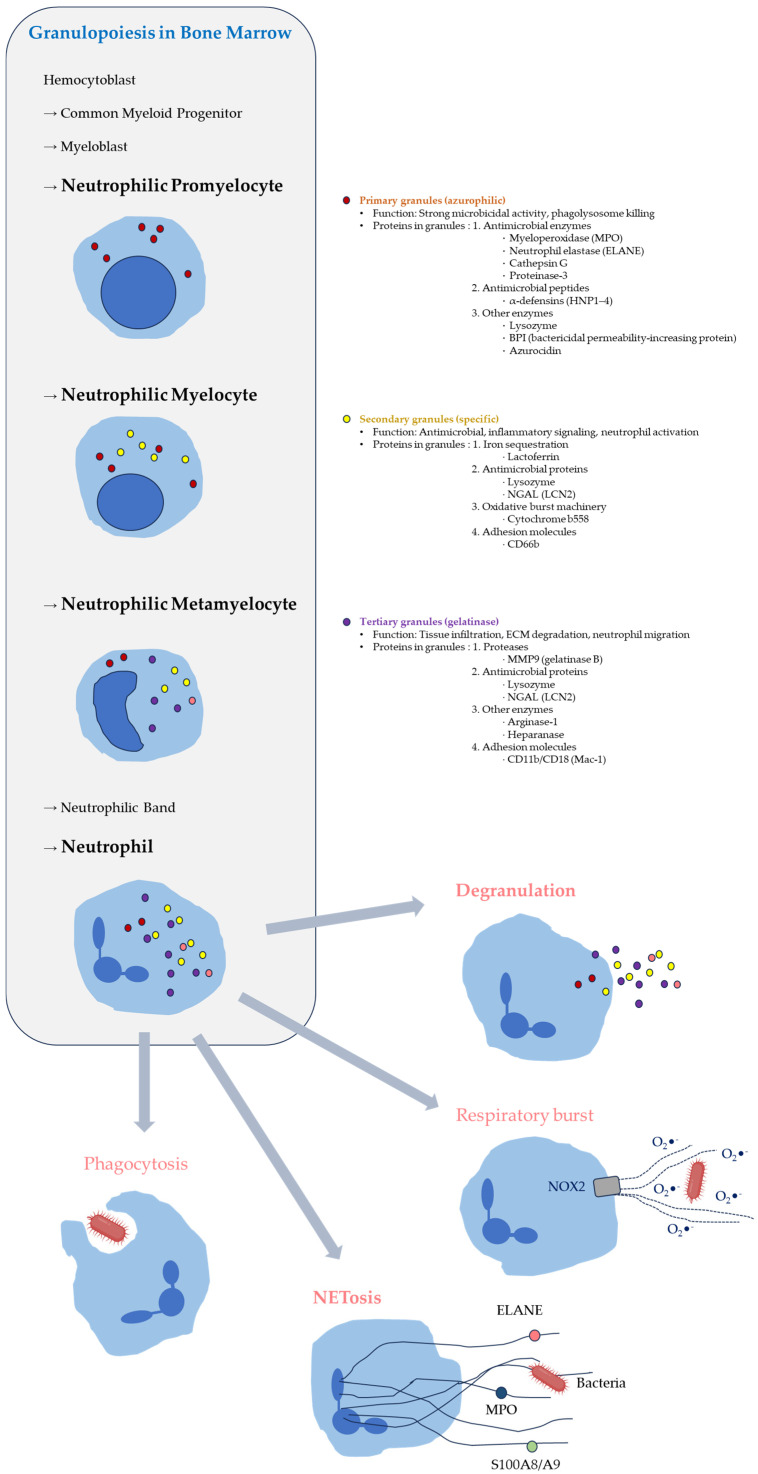
Granulopoiesis and antimicrobial effector mechanisms of neutrophils. Neutrophils are produced in the bone marrow through a stepwise process of granulopoiesis, progressing from hematopoietic progenitors to mature segmented neutrophils. During neutrophil differentiation, distinct granule subsets are generated sequentially: primary (azurophilic) granules during the promyelocyte stage, secondary (specific) granules during the myelocyte stage, and tertiary (gelatinase) granules during the metamyelocyte stage. These granules store diverse antimicrobial enzymes and inflammatory mediators that are preloaded prior to the release of mature neutrophils into circulation. Upon activation at sites of infection or inflammation, neutrophils deploy several rapid effector mechanisms. These include phagocytosis of invading microbes, degranulation-mediated release of antimicrobial proteins such as myeloperoxidase (MPO) and neutrophil elastase (ELANE), formation of neutrophil extracellular traps (NETosis), and the generation of reactive oxygen species through the NADPH oxidase complex (NOX2), leading to a respiratory burst that contributes to microbial killing. Together, these preformed effector systems enable neutrophils to mount rapid antimicrobial responses without requiring de novo protein synthesis.

Understanding this phenomenon requires consideration not only of calprotectin’s molecular functions but also of the broader biological context of neutrophil differentiation and effector programming. During granulopoiesis, neutrophils undergo a highly regulated transcriptional program that leads to the synthesis and storage of large quantities of antimicrobial and inflammatory proteins prior to their release into the bloodstream [18,38,39] (Figure 1). This process results in a distinctive cellular architecture in which the mature neutrophil is equipped with a preassembled effector proteome optimized for rapid deployment during infection.

Within this framework, the marked accumulation of S100A8/A9 may represent a specialized adaptation of neutrophils that integrates antimicrobial defense, metal homeostasis, inflammatory signaling, and cellular stress regulation.

In this review, we examine the high abundance of S100A8/A9 within the neutrophil cytosolic proteome and explore the possible biological logic underlying this phenomenon. We first summarize the composition of the neutrophil cytosolic proteome and the developmental programs that drive selective expression of neutrophil effector proteins. We then review the structural and functional properties of S100A8/A9 and evaluate the major hypotheses that have been proposed to explain their extraordinary abundance.

Finally, we discuss the evolutionary and immunological implications of calprotectin enrichment in neutrophils and highlight key unanswered questions that may guide future research into the cellular strategy of neutrophil effector protein preloading.

**Table 1:** Proposed functional hypotheses explaining the high intracellular abundance of S100A8/A9 (calprotectin) in neutrophils.

Functional Role	Mechanistic Basis	Biological Context	Implication for Abundance	Key Reference
Metal sequestration-mediated antimicrobial activity (nutritional immunity)	High-affinity binding of Zn^2+^ and Mn^2+^ by S100A8/A9 limits microbial access to essential metals	Host–pathogen competition for essential metals during infection	High intracellular concentrations enable efficient sequestration of essential metal ions and facilitate rapid antimicrobial activity at sites of infection	[32,33,34,35,40,41]
Regulation of inflammatory signaling	Activation of pattern-recognition receptors including TLR4 and RAGE by extracellular S100A8/A9	Acute and chronic inflammation	High intracellular abundance ensures strong release of inflammatory mediators during neutrophil activation or cell death	[29,30,31]
Oxidative stress modulation	Interaction with NADPH oxidase components and modulation of reactive oxygen species production	Neutrophil antimicrobial responses	High concentrations may buffer oxidative stress generated during respiratory burst	[30,42]
Cytosolic metal and protein buffering	Binding of transition metals and interactions with cytoskeletal proteins influencing intracellular homeostasis	Neutrophil activation and migration	High cytosolic abundance may stabilize intracellular metal availability and cellular stress responses	[21,36,37]
Damage-associated molecular pattern (DAMP) signaling	Release of S100A8/A9 from activated or dying neutrophils activates innate immune receptors	Tissue injury and inflammatory amplification	Neutrophils may serve as reservoirs of DAMP molecules enabling rapid inflammatory signaling	[29,31,43]
Tumor microenvironment regulation	Recruitment of myeloid cells and promotion of pre-metastatic niche formation	Cancer progression and metastasis	Abundant neutrophil-derived S100A8/A9 may influence tumor-associated inflammation and metastatic colonization	[21,44,45]

## 2. The Cytosolic Proteome of Neutrophils

### 2.1. Global Organization of the Neutrophil Cytosolic Proteome

Neutrophils exhibit a proteomic architecture optimized for rapid innate immune responses. Mature neutrophils rely on a preassembled repertoire of proteins synthesized during granulopoiesis in the bone marrow, resulting in a cytoplasm enriched in antimicrobial proteins, inflammatory mediators, and enzymes required for immediate host defense [1,3,4,8,11].

**Figure 2:**
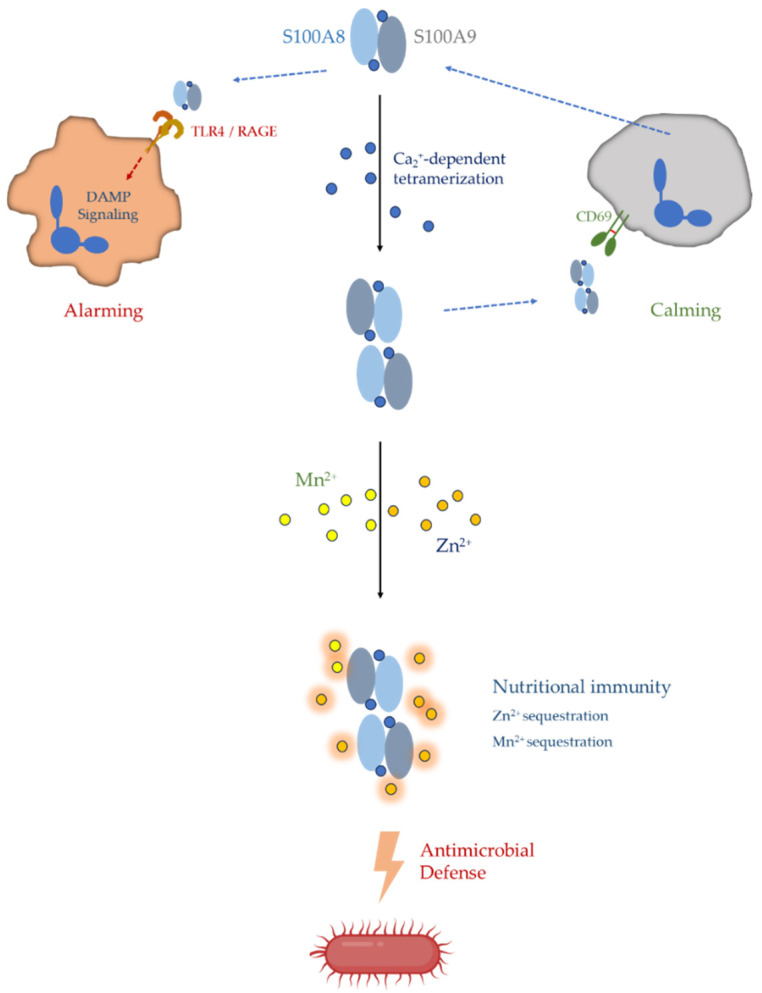
High abundance of calprotectin and its functional mechanisms in neutrophils. Calprotectin, a heterodimer composed of S100A8 and S100A9, is a prominent protein complex in the neutrophil cytosol. This unusual abundance reflects the unique biochemical specialization of neutrophils as preloaded effector cells in innate immunity. Upon activation or cellular damage, S100A8/A9 can be released extracellularly and function as a damage-associated molecular pattern (DAMP), engaging pattern-recognition receptors such as Toll-like receptor 4 (TLR4) and the receptor for advanced glycation end products (RAGE) to promote inflammatory signaling. Calcium binding induces Ca^2+^-dependent tetramerization of the S100A8/A9 complex, generating high-affinity transition metal-binding sites capable of sequestering essential microbial nutrients such as Zn^2+^ and Mn^2+^. Through this process of nutritional immunity, calprotectin restricts microbial access to critical metal ions and contributes to antimicrobial defense. In addition to its proinflammatory alarmin functions, S100A8/A9 can also participate in regulatory pathways that contribute to the resolution of inflammation.

Large-scale proteomic analyses show that the neutrophil cytoplasm is dominated by a limited number of highly abundant proteins, creating a highly skewed proteomic distribution (Figure 2). These proteins primarily include cytoskeletal components, glycolytic enzymes, and neutrophil-specific antimicrobial proteins [11,22,46].

Neutrophils rely predominantly on glycolysis for ATP generation and have relatively limited mitochondrial capacity, reflecting their specialization for rapid effector responses [1,9,47].

During granulopoiesis, neutrophils sequentially synthesize antimicrobial proteins that are packaged into primary, secondary, and tertiary granules [9,11,48,49,50]. These granules contain diverse antimicrobial peptides, proteases, and enzymes and are rapidly mobilized through regulated exocytosis upon activation, enabling efficient deployment of antimicrobial molecules during infection and inflammation [47,51,52,53].

In contrast, S100A8 and S100A9 remain predominantly cytosolic rather than being stored in granules, distinguishing them from most neutrophil antimicrobial proteins [1,49,50]. While granule proteins constitute a major fraction of total cellular protein, the cytosolic compartment remains disproportionately enriched in S100A8/A9, highlighting a distinct mode of proteomic organization. This localization suggests that calprotectin may serve broader cytosolic and extracellular regulatory functions beyond classical granule-associated antimicrobial activity.

Recent quantitative proteomic analyses and single-cell transcriptomic studies have shown that neutrophils are characterized by a highly skewed molecular composition dominated by a limited number of highly expressed genes and proteins, with S100A8/A9 consistently among the most abundant components [22,23].

### 2.2. Enrichment of Calprotectin in the Neutrophil Cytosol

Among the proteins present in the neutrophil cytosol, the calcium-binding proteins S100A8 and S100A9 are among the most prominent components. Early biochemical studies estimated that S100A8/A9 constitutes a substantial fraction of the neutrophil cytosolic proteome [20,21]. These proteins exist primarily as heterodimeric complexes known as calprotectin and are present at extremely high intracellular concentrations. The predominance of this complex within the cytosolic proteome distinguishes neutrophils from most other leukocytes and suggests a fundamental role in neutrophil biology. These observations are consistent with both early biochemical measurements and recent high-throughput proteomic analyses, reinforcing the robust enrichment of calprotectin in neutrophils.

The unusual abundance of calprotectin becomes even more striking when compared with the typical abundance of signaling proteins in immune cells. In most cellular systems, signaling mediators are present at relatively low concentrations and exert their effects through amplification cascades. Structural proteins and metabolic enzymes, by contrast, often dominate cellular proteomes because they are required in large quantities to maintain cellular architecture or sustain metabolic flux. S100A8/A9, however, does not fall neatly into either of these categories. Although these proteins participate in inflammatory signaling and antimicrobial defense, their massive representation in neutrophils far exceeds the levels typically required for conventional signaling functions [21,25,29].

Taken together, these observations highlight a fundamental and unresolved paradox in neutrophil biology. The neutrophil cytosol is strongly enriched by S100A8/A9, yet the biological rationale for such unusual abundance remains incompletely understood. While numerous studies have examined the antimicrobial and inflammatory activities of calprotectin, relatively little attention has been devoted to explaining why neutrophils allocate such a large fraction of their cytosolic proteome to this single protein complex [21,42,43].

Resolving this question requires consideration not only of calprotectin function but also of the developmental programs that shape neutrophil protein expression during granulopoiesis. In the following section, we therefore examine the transcriptional and developmental mechanisms that drive selective expression of neutrophil effector proteins, with particular emphasis on the regulatory programs that govern the remarkable production of S100A8 and S100A9 during neutrophil differentiation [11,54,55].

## 3. Transcriptional Programming of Neutrophil Effector Proteins

### 3.1. Granulopoiesis and Stage-Specific Protein Expression

The remarkable abundance of specific proteins in neutrophils cannot be understood solely from the perspective of protein function. Instead, it reflects the distinctive developmental program that governs neutrophil differentiation in the bone marrow. Neutrophils arise through a tightly regulated process of granulopoiesis in which hematopoietic stem cells progressively differentiate into mature polymorphonuclear leukocytes through multiple intermediate stages. During this process, large quantities of effector proteins are synthesized and stored before the cells enter circulation, resulting in the characteristic preloaded proteome of mature neutrophils [1,3,8,39,56].

Granulopoiesis proceeds through a series of morphologically and transcriptionally distinct stages, including myeloblasts, promyelocytes, myelocytes, metamyelocytes, band cells, and finally mature neutrophils. Importantly, the expression of many neutrophil effector proteins is tightly coupled to specific stages of this differentiation process. Early in granulopoiesis, promyelocytes synthesize proteins that are subsequently packaged into primary granules, including myeloperoxidase and defensins. At later stages, additional classes of proteins such as lactoferrin are produced and incorporated into secondary granules [9,48,49,50,57].

This sequential pattern of gene expression, often referred to as the “targeting-by-timing” model, ensures that proteins synthesized at particular stages of differentiation are directed into the appropriate intracellular compartments [9,57]. Such developmental programming allows neutrophils to accumulate large quantities of antimicrobial proteins during their maturation in the bone marrow. By the time neutrophils enter circulation, they are already equipped with a substantial arsenal of effector molecules capable of immediate deployment upon encountering pathogens or inflammatory signals [2,11,12,38].

Unlike many granule proteins, however, S100A8 and S100A9 are not primarily stored within granules but remain largely in the cytosol of mature neutrophils. Their expression increases dramatically during the later stages of granulopoiesis, particularly at the myelocyte and metamyelocyte stages. This stage-specific expression pattern suggests that the accumulation of S100A8/A9 is driven by transcriptional programs associated with terminal neutrophil differentiation rather than by immediate inflammatory stimuli [11,54,55].

### 3.2. Transcriptional Regulators of Neutrophil Differentiation

The transcriptional regulation of neutrophil differentiation is orchestrated by a network of lineage-defining transcription factors that control the expression of neutrophil-specific genes. Among these, members of the CCAAT/enhancer-binding protein (C/EBP) family play particularly central roles in granulocytic development [58,59].

C/EBPα is required for the early stages of granulocytic differentiation and promotes the commitment of hematopoietic progenitors to the granulocyte lineage. Disruption of C/EBPα activity leads to impaired granulopoiesis and a block in neutrophil differentiation [59,60,61]. As neutrophil maturation proceeds, C/EBPε becomes increasingly important for the regulation of genes associated with terminal neutrophil differentiation, including those encoding several antimicrobial and inflammatory proteins [49,58,61].

Additional transcription factors further refine the neutrophil gene expression program. PU.1, a member of the ETS (E26 transformation-specific) family of transcription factors, regulates numerous genes involved in myeloid lineage development and contributes to both macrophage and neutrophil differentiation [39,56,62]. GFI1 acts as a transcriptional repressor that promotes neutrophil maturation by suppressing alternative lineage programs and stabilizing granulocytic differentiation pathways [54,62,63,64].

Single-cell transcriptomic analyses have further revealed that myeloid progenitors display substantial transcriptional heterogeneity prior to terminal neutrophil differentiation, indicating that lineage commitment involves progressive restriction of developmental potential [54,65].

Together, these transcription factors establish the regulatory network that defines the neutrophil lineage and coordinates the large-scale production of effector proteins required for host defense. Through the coordinated action of these regulatory factors, neutrophil progenitors undergo a transcriptional program that strongly favors the expression of a relatively limited set of highly abundant proteins [49,54,65].

### 3.3. Epigenetic Regulation and Post-Differentiation Constraints

Beyond transcription factor networks, chromatin organization and epigenetic regulation also contribute to shaping neutrophil gene expression. Genome-wide analyses of chromatin accessibility have shown that neutrophil differentiation is accompanied by the progressive activation of enhancer elements associated with inflammatory and antimicrobial genes [54,55,66]. These regulatory regions facilitate high levels of transcription during granulopoiesis and support the large-scale production of proteins that will later populate the neutrophil cytosol and granules.

Additional studies of hematopoietic lineage commitment have shown that transcriptional priming of myeloid progenitors precedes terminal differentiation and may predispose cells toward specific immune effector programs [66,67]. Interferon regulatory factor pathways and other transcriptional regulators can also influence myeloid lineage responses during immune activation [68].

Another important feature of neutrophil biology is the dramatic reduction in transcriptional capacity that occurs after terminal differentiation. Mature circulating neutrophils exhibit relatively limited transcriptional activity compared with other leukocyte populations. This reduced transcriptional activity reflects both their short lifespan and their specialization as immediate effector cells [12,13,15].

Consequently, the functional capabilities of neutrophils depend heavily on proteins synthesized earlier during bone marrow maturation. The marked accumulation of effector proteins during granulopoiesis therefore represents a critical component of neutrophil immune strategy [1,3,11]. Within this framework, the remarkable abundance of S100A8 and S100A9 can be viewed as a product of developmental programming rather than solely a response to environmental stimuli.

The transcriptional networks governing granulopoiesis appear to favor the robust expression of a relatively small set of effector proteins, resulting in a cytosolic proteome dominated by a limited number of highly abundant molecules. Such a strategy may represent an evolutionary adaptation that prioritizes rapid antimicrobial activity and inflammatory signaling during the earliest phases of host defense [8,11,13].

Understanding why S100A8/A9 dominates the neutrophil cytosol therefore requires integrating knowledge of transcriptional programming with the biochemical and functional properties of these proteins. In the following section, we examine the structural and biochemical features of S100A8 and S100A9 that may help explain their unique role in neutrophil biology and their remarkable accumulation within the cytosol.

## 4. Structural and Biochemical Properties of S100A8/A9

### 4.1. Structural Organization of the S100A8/A9 Complex

S100A8 and S100A9 belong to the S100 family of calcium-binding proteins, a group of small acidic proteins characterized by the presence of EF-hand calcium-binding motifs. Members of the S100 family are typically 10–12 kDa in size and function primarily as intracellular calcium sensors that undergo conformational changes upon binding Ca^2+^, thereby enabling interactions with various target proteins. S100A8 and S100A9 are encoded within the S100 gene cluster located on chromosome 1q21, a genomic region that contains multiple genes associated with inflammatory and epithelial functions [24,25,69].

A distinctive feature of S100A8 and S100A9 is their tendency to form stable heterodimeric complexes. In neutrophils, the two proteins associate predominantly as a non-covalent heterodimer known as calprotectin. Structural studies have demonstrated that each monomer contains two EF-hand domains: a canonical C-terminal EF-hand with high affinity for calcium and a non-canonical N-terminal EF-hand with lower affinity. Binding of calcium induces conformational rearrangements that expose hydrophobic surfaces on the protein complex, thereby enabling interactions with other proteins and cellular components [27,28,70,71].

**Figure 3:**
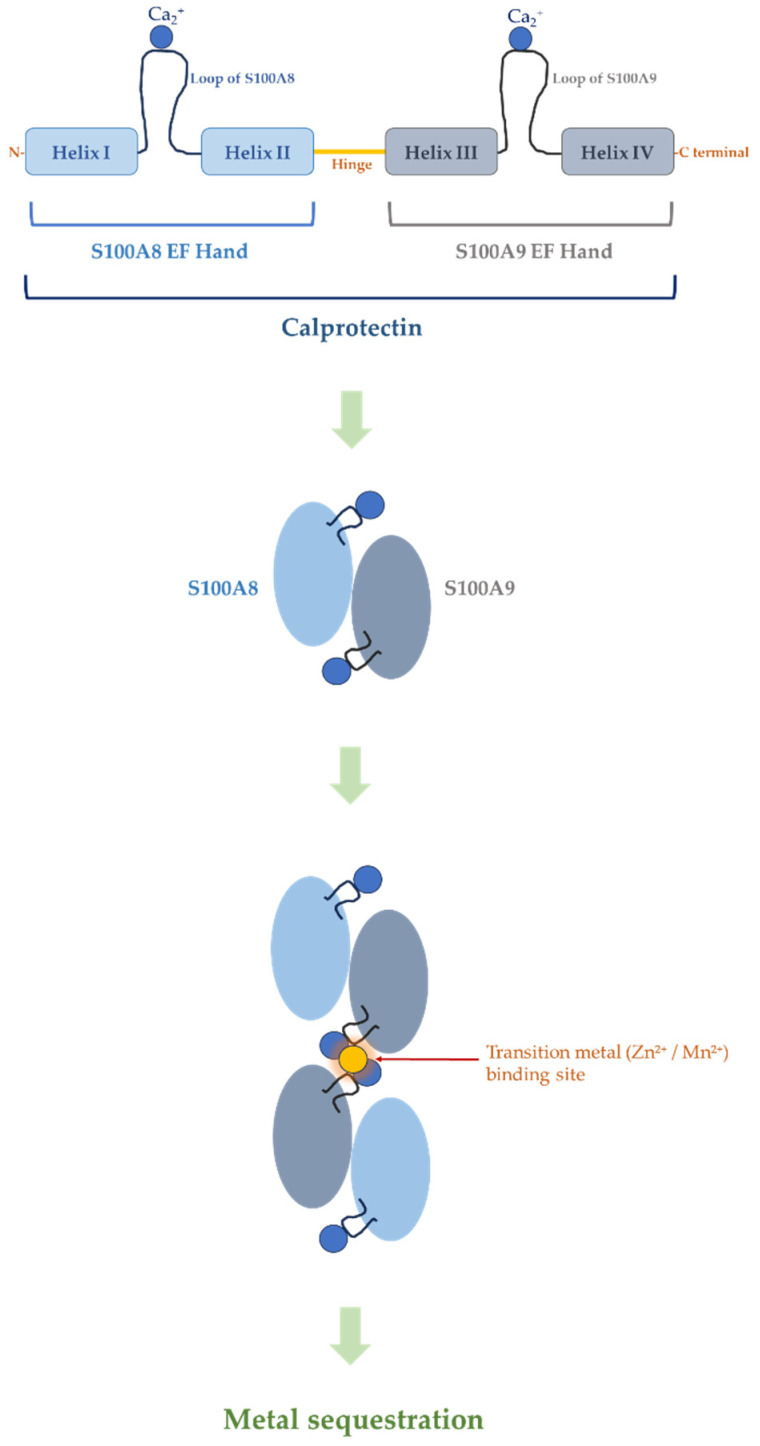
Structural organization of the S100A8/A9 (calprotectin) complex and its transition metal-binding mechanism. Each S100 subunit contains EF-hand motifs that bind Ca^2+^, promoting conformational changes and stabilization of the S100A8/A9 heterodimer. Calcium binding further supports the assembly of higher-order complexes, including tetramers. At the tetramer interface, transition metal-binding sites are formed that enable the sequestration of Zn^2+^ and Mn^2+^. These sites are generated through histidine-rich coordination environments contributed by multiple subunits, allowing calprotectin to bind transition metals through a mechanism distinct from canonical EF-hand calcium binding. This process contributes to metal sequestration and antimicrobial defense.

Under physiological conditions, the S100A8/A9 heterodimer can further assemble into higher-order oligomeric structures. In particular, calcium binding promotes the formation of heterotetramers consisting of two S100A8/A9 heterodimers. This oligomerization state is thought to stabilize the protein complex and may influence its interactions with cellular membranes, cytoskeletal components, and extracellular receptors [27,40,42,70]. These structural features are schematically illustrated in Figure 3. The ability of calprotectin to adopt multiple oligomeric states suggests a dynamic structural framework that may allow the protein to participate in diverse intracellular and extracellular processes.

### 4.2. Transition Metal Binding and Nutritional Immunity

Beyond calcium binding, one of the most remarkable biochemical properties of S100A8/A9 is its ability to bind transition metals with high affinity. Structural and biochemical analyses have shown that calprotectin forms high-affinity metal-binding sites at the interface between the two subunits, enabling the protein complex to coordinate divalent metal ions such as zinc and manganese [40,41,72].

Importantly, the mechanism of transition metal binding by S100A8/A9 differs fundamentally from calcium coordination by EF-hand motifs. While Ca^2+^ is coordinated within canonical EF-hand loops primarily through oxygen-donor ligands such as aspartate and glutamate residues, the binding of transition metals such as Zn^2+^ and Mn^2+^ occurs at the interface of the S100A8/A9 tetramer and involves distinct coordination chemistry. Structural studies have shown that these transition metal-binding sites are formed by histidine-rich motifs and additional coordinating residues contributed by multiple subunits, generating high-affinity metal-binding pockets. This interface-driven coordination allows calprotectin to effectively sequester transition metals with different chemical properties compared with calcium, thereby expanding its functional repertoire beyond classical calcium signaling [35,40,41]. Subsequent studies have expanded this concept by demonstrating that calprotectin can also bind additional metal ions, including ferrous iron (Fe^2+^), further enhancing its antimicrobial capacity [35,73,74].

This metal-binding capacity underlies one of the most well-established functions of calprotectin in innate immunity. By sequestering essential transition metals, calprotectin restricts microbial access to nutrients required for bacterial metabolism and growth. This host defense strategy is commonly referred to as nutritional immunity and represents an important mechanism by which the immune system suppresses microbial proliferation [33,34,35].

Experimental studies have demonstrated that calprotectin can inhibit the growth of numerous bacterial and fungal pathogens by depriving them of essential metal cofactors [32,33,34]. Because many microbial enzymes depend on zinc and manganese as essential cofactors for catalytic activity, including metalloproteases, superoxide dismutase, and enzymes involved in DNA synthesis and central metabolism, the ability of calprotectin to chelate these metals can severely impair microbial metabolic processes. In infected tissues, neutrophil-derived calprotectin therefore contributes to the establishment of an antimicrobial environment that limits pathogen expansion [32,33,35].

The high abundance of S100A8/A9 within neutrophils may therefore provide a large reservoir of metal-binding capacity that can be rapidly deployed upon neutrophil activation or cell death. When neutrophils release calprotectin into the extracellular space, the resulting high local concentrations of the protein can effectively compete with microbial metal acquisition systems, thereby reinforcing host defense mechanisms [21,34,35].

### 4.3. Intracellular Functions and Functional Versatility

Another notable biochemical characteristic of S100A8/A9 is its unusual intracellular concentration in neutrophils. Measurements of cytosolic protein composition have shown that calprotectin reaches concentrations far exceeding those typical of signaling proteins or regulatory factors [20,21]. Such high abundance suggests that the protein may function not only as a signaling molecule but also as a molecular buffer capable of interacting with metal ions, reactive oxygen species, and other cellular components at high capacity.

In addition to its extracellular antimicrobial role, several studies have suggested that S100A8/A9 participates in intracellular regulatory processes within neutrophils. The protein complex has been reported to interact with cytoskeletal elements and may influence neutrophil migration and adhesion [21,29,75].

The biochemical versatility of S100A8/A9 also enables interactions with a variety of intracellular targets involved in inflammatory signaling and cellular stress responses. In addition, extracellular S100A8/A9 released from activated or dying neutrophils can function as a damage-associated molecular pattern (DAMP) molecule capable of activating pattern-recognition receptors such as Toll-like receptor 4 and the receptor for advanced glycation end products [21,29,31].

Taken together, the structural and biochemical characteristics of S100A8/A9 reveal a multifunctional protein complex capable of sensing calcium signals, binding transition metals, and interacting with diverse cellular targets. These properties provide a mechanistic foundation for the various antimicrobial and inflammatory activities attributed to calprotectin [24,25,69].

However, while these functions help explain how S100A8/A9 participates in host defense, they do not fully account for the unusual enrichment of this protein complex within the neutrophil cytosol. Understanding why neutrophils accumulate such massive quantities of calprotectin therefore requires examining the functional hypotheses that have been proposed to explain this unusual biological phenomenon.

## 5. Why Is Calprotectin So Abundant in Neutrophils?

Despite extensive research on the biological functions of S100A8 and S100A9, the remarkable enrichment of this protein complex within the neutrophil cytosol remains difficult to explain. The massive intracellular accumulation of calprotectin suggests that its abundance must provide a substantial functional advantage to neutrophils during host defense. Several non-mutually exclusive hypotheses have therefore been proposed to explain why neutrophils devote such a large fraction of their cytosolic proteome to S100A8/A9. These hypotheses generally relate to antimicrobial defense, metal homeostasis, inflammatory signaling, and cellular stress regulation [20,21,25,26,29].

### 5.1. Antimicrobial Reservoir Hypothesis

One of the most widely discussed explanations for the abundance of S100A8/A9 in neutrophils is that calprotectin functions as a large intracellular reservoir of antimicrobial molecules that can be rapidly released during infection or inflammation. Activated neutrophils are capable of releasing large quantities of cytosolic proteins through mechanisms such as cell lysis, NET formation, or inflammatory cell death [3,7,76].

Because S100A8/A9 is present at extremely high intracellular concentrations, its release into infected tissues can rapidly generate local concentrations sufficient to exert antimicrobial effects. Experimental studies have demonstrated that calprotectin can inhibit the growth of numerous bacterial and fungal pathogens. The antimicrobial activity of the protein complex is largely attributed to its ability to deprive microorganisms of essential metal ions required for metabolic processes [32,33,34,77,78].

Further evidence supporting this hypothesis comes from studies showing that calprotectin is present within neutrophil extracellular traps (NETs), where it contributes to antimicrobial defense against pathogens such as *Candida albicans* [76].

Within this framework, neutrophils may function as mobile reservoirs of antimicrobial proteins that can be deployed immediately at sites of infection. The unusually large intracellular pool of calprotectin would therefore ensure that neutrophils can rapidly establish antimicrobial conditions in infected tissues [21,34,79,80].

### 5.2. Metal Sequestration and Nutritional Immunity

A closely related explanation for calprotectin abundance centers on its role in metal sequestration during host defense. Many microbial pathogens require transition metals such as zinc and manganese as cofactors for essential enzymatic reactions. To counter this requirement, the host immune system employs mechanisms that limit microbial access to these metals, a process known as nutritional immunity [33,34].

Calprotectin plays a central role in this defense strategy by binding transition metals with high affinity. Structural studies have demonstrated that the S100A8/A9 heterodimer forms specialized metal-binding sites capable of coordinating zinc and manganese ions [40,41]. Through this mechanism, calprotectin effectively competes with microbial metal acquisition systems.

The extremely high abundance of S100A8/A9 in neutrophils may therefore maximize the capacity of the immune system to sequester transition metals during infection. Upon release from neutrophils, the large extracellular concentration of calprotectin can significantly reduce the availability of essential metals in the local microenvironment, thereby inhibiting microbial growth [21,32,34,80].

### 5.3. Regulation of Reactive Oxygen Species and Redox Homeostasis

Another hypothesis proposes that S100A8/A9 contributes to the regulation of oxidative stress within neutrophils. Neutrophils generate large quantities of reactive oxygen species (ROS) during the oxidative burst, which plays a central role in microbial killing. However, excessive ROS production can also damage host cellular components [2,47].

Several studies have suggested that S100A8/A9 may modulate the activity of the NADPH oxidase complex responsible for ROS production [30]. In addition, S100A8/A9 proteins can undergo oxidative modifications, suggesting that they may participate in cellular redox reactions [42,81].

If calprotectin contributes to buffering oxidative stress within neutrophils, its high intracellular abundance could help stabilize the intracellular environment during periods of intense antimicrobial activity. In this view, the marked accumulation of S100A8/A9 may represent a protective adaptation that allows neutrophils to tolerate the extreme oxidative conditions associated with pathogen killing [21,29].

### 5.4. Cytosolic Protein and Metal Buffering

A further possibility is that S100A8/A9 functions as a cytosolic buffering protein that helps maintain intracellular homeostasis. The extremely high concentration of calprotectin suggests that it may influence the biochemical properties of the neutrophil cytoplasm, including the availability of metal ions and interactions with other proteins [21,42,82].

Some studies have reported interactions between S100A8/A9 and cytoskeletal components, suggesting potential roles in regulating neutrophil migration and cellular dynamics [30,36,37]. In addition, the metal-binding capacity of calprotectin may allow it to function as an intracellular metal buffer that stabilizes fluctuations in transition metal concentrations.

Although the buffering hypothesis remains less well established than the antimicrobial or nutritional immunity models, it highlights the possibility that calprotectin abundance may support intracellular regulatory functions in addition to extracellular antimicrobial activity [25,29].

### 5.5. Calprotectin as a Reservoir of Damage-Associated Molecular Patterns

Finally, the remarkable abundance of S100A8/A9 may reflect its function as a major source of damage-associated molecular patterns (DAMPs) released during inflammation. When neutrophils undergo activation, necrosis, or NET formation, large quantities of cytosolic proteins are released into the extracellular environment [3,7,76].

Extracellular S100A8/A9 can act as endogenous danger signals that activate pattern-recognition receptors such as Toll-like receptor 4 and the receptor for advanced glycation end products. Through these interactions, calprotectin can amplify inflammatory responses by promoting cytokine production and leukocyte recruitment [29,30,31,83,84,85].

In this context, neutrophils may function as reservoirs of inflammatory mediators capable of rapidly initiating and amplifying innate immune responses. However, although the DAMP function of S100A8/A9 is well-documented, it remains unclear whether this role alone can explain the massive intracellular accumulation of the protein [21,25,82].

### 5.6. Integrative Perspective: A Multifunctional Effector Strategy

Taken together, these hypotheses suggest that the remarkable abundance of calprotectin in neutrophils may reflect the convergence of multiple functional advantages rather than a single dominant mechanism. S100A8/A9 combines several properties that are particularly useful for neutrophil biology, including antimicrobial activity, metal sequestration, regulation of oxidative stress, and inflammatory signaling [21,25,29,69].

The evolutionary selection of such a multifunctional protein complex may therefore represent an efficient strategy for equipping neutrophils with a versatile molecular toolkit capable of supporting diverse aspects of innate immune defense [26,82].

## 6. Evolutionary Logic of Neutrophil Protein Preloading

### 6.1. Neutrophils as Preloaded Effector Cells

Neutrophils are uniquely adapted for rapid antimicrobial defense and represent one of the earliest cellular responders to infection. Unlike lymphocytes and many other immune cells that rely heavily on inducible transcriptional responses following activation, neutrophils function primarily as preloaded effector cells containing large quantities of antimicrobial and inflammatory proteins synthesized during granulopoiesis in the bone marrow [1,3,8,11].

This developmental strategy reflects the biological constraints imposed on mature neutrophils. Circulating neutrophils have a relatively short lifespan and exhibit limited transcriptional activity compared with many other leukocyte populations. Classical studies of neutrophil turnover demonstrated rapid production and clearance kinetics, highlighting the transient nature of these cells in circulation [12,14]. More recent in vivo labeling studies have refined these estimates and confirmed the tightly regulated lifespan of circulating neutrophils [15]. As a consequence, neutrophils rely heavily on proteins that have already been synthesized during earlier stages of differentiation. The functional capabilities of mature neutrophils therefore depend largely on the intracellular stores of effector molecules accumulated during granulopoiesis [11,13,38].

During neutrophil maturation, large quantities of antimicrobial enzymes, inflammatory mediators, and cytotoxic proteins are synthesized and stored within the cell. Granule proteins such as myeloperoxidase, defensins, and lactoferrin are produced at defined stages of granulopoiesis and subsequently packaged into specific granule compartments. Upon neutrophil activation, these proteins can be rapidly released through degranulation or during the formation of neutrophil extracellular traps [9,48,76].

Cytosolic proteins such as S100A8 and S100A9 represent a complementary class of effector molecules that are not confined to granules but remain available within the cytoplasm for rapid release or intracellular activity. Because these proteins are present at extremely high concentrations within neutrophils, their release during inflammation can rapidly generate high extracellular concentrations capable of influencing the surrounding immune environment [20,21,29].

The preloading strategy of neutrophils therefore represents an evolutionary adaptation that prioritizes immediate functional capacity over transcriptional flexibility. By synthesizing large quantities of effector proteins during development, neutrophils are able to respond rapidly to microbial invasion without the delay associated with inducible gene expression [7,8,86].

### 6.2. Evolutionary Advantages of Calprotectin Enrichment

Within this framework, the remarkable abundance of S100A8/A9 may reflect an evolutionary strategy that favors multifunctional proteins capable of supporting several aspects of innate immune defense simultaneously. Calprotectin possesses antimicrobial activity, metal-binding capacity, inflammatory signaling properties, and potential intracellular regulatory functions [21,25,29]. The accumulation of large quantities of this protein complex may therefore provide neutrophils with a versatile molecular resource capable of addressing multiple challenges encountered during infection.

One possible advantage of such a strategy is biosynthetic efficiency. Producing a limited set of highly abundant multifunctional proteins may be metabolically advantageous compared with synthesizing a large number of specialized proteins. In this sense, calprotectin may function as a molecular “Swiss army knife,” capable of performing several defensive roles while minimizing the complexity of the neutrophil proteome [21,25].

Another potential advantage relates to the environmental conditions in which neutrophils operate. Neutrophils are often recruited to sites of infection characterized by high microbial burden, intense inflammatory signaling, and significant cellular stress. Under these conditions, the rapid release of large quantities of antimicrobial and regulatory proteins may be essential for controlling pathogen proliferation during the earliest stages of immune defense [7,33,34].

The enrichment of S100A8/A9 within the neutrophil cytosol may therefore represent an example of proteomic prioritization, in which the cellular proteome is strongly biased toward a small number of proteins that provide maximal functional benefit during acute immune responses. From this perspective, calprotectin may represent one of the most extreme examples of proteomic specialization within the innate immune system [21,29,42].

### 6.3. Proteomic Prioritization in Innate Immune Cells

The concept of proteomic prioritization may provide a useful framework for understanding the unusual abundance of calprotectin in neutrophils. In many cell types, protein expression levels are distributed relatively evenly across a wide range of proteins with specialized functions. In contrast, neutrophils appear to concentrate a substantial fraction of their proteome in a limited number of highly abundant effector molecules [22,46].

Recent proteomic studies have further shown that neutrophil effector proteins can undergo dynamic remodeling during inflammation, indicating that the neutrophil proteome is both highly specialized and tightly regulated [87].

Such a strategy may reflect the evolutionary pressures faced by innate immune cells that must respond rapidly to unpredictable microbial threats. Rather than maintaining a complex and highly regulated proteome, neutrophils may instead rely on a simplified but highly concentrated set of effector proteins capable of performing multiple defensive functions [3,7,8,86].

Within this context, apparent enrichment of S100A8/A9 in neutrophils may represent a central element of this proteomic strategy. The extraordinary abundance of calprotectin ensures that neutrophils possess a large reservoir of molecules capable of participating in antimicrobial defense, metal sequestration, inflammatory signaling, and cellular stress regulation [21,25,29].

Although the precise evolutionary forces responsible for this proteomic configuration remain uncertain, the persistence of extremely high S100A8/A9 expression across mammalian species suggests that this system confers a significant selective advantage in host defense [25,26,29].

## 7. Implications for Disease and the Tumor Microenvironment

### 7.1. Calprotectin in Inflammatory Diseases

The remarkable abundance of S100A8/A9 in neutrophils has important implications for a wide range of inflammatory diseases. Because neutrophils are rapidly recruited to sites of tissue injury and infection, the large intracellular reservoir of calprotectin represents a potent source of inflammatory mediators that can shape local immune responses. Upon neutrophil activation, necrosis, or the formation of neutrophil extracellular traps (NETs), substantial amounts of S100A8/A9 are released into the extracellular environment [3,7,76].

Extracellular calprotectin functions as a damage-associated molecular pattern (DAMP) capable of activating innate immune receptors. In particular, S100A8/A9 has been shown to interact with pattern-recognition receptors such as Toll-like receptor 4 (TLR4) and the receptor for advanced glycation end products (RAGE), thereby initiating downstream inflammatory signaling pathways [29,30,31]. Through these interactions, calprotectin can promote cytokine production, leukocyte recruitment, and amplification of local inflammatory responses [43,88].

Elevated levels of S100A8/A9 have been reported in numerous inflammatory disorders, including rheumatoid arthritis, psoriasis, and inflammatory bowel disease [20,21,89,90]. In these conditions, activated neutrophils and inflammatory monocytes release calprotectin into tissues and body fluids, contributing to sustained inflammatory signaling [28,91,92].

One of the most widely used clinical applications of calprotectin measurement is the assessment of intestinal inflammation. Fecal calprotectin has become a well-established biomarker for inflammatory bowel disease and is commonly used in clinical practice to distinguish inflammatory bowel disease from functional gastrointestinal disorders and to monitor disease activity [93,94,95,96]. The diagnostic value of fecal calprotectin is largely attributable to the extremely high intracellular concentration of S100A8/A9 in neutrophils and the relative stability of the protein complex in extracellular environments [31].

These observations illustrate how the massive intracellular reservoir of calprotectin in neutrophils can translate into significant extracellular signaling effects during inflammatory disease.

### 7.2. Calprotectin in the Tumor Microenvironment

In addition to its role in classical inflammatory diseases, calprotectin has emerged as an important mediator within the tumor microenvironment. Many tumors recruit large numbers of myeloid cells, including neutrophils and myeloid-derived suppressor cells (MDSCs), which can release S100A8/A9 into the surrounding tissue [8,29,97].

Within tumors, S100A8/A9 can influence several processes associated with tumor progression. These proteins have been shown to promote the recruitment and accumulation of myeloid cells within tumors, thereby contributing to the establishment of an inflammatory microenvironment that supports tumor growth [45,98,99].

In addition, S100A8/A9 can interact with receptors such as RAGE and TLR4 on tumor cells and immune cells, activating signaling pathways that influence cell survival, proliferation, and migration [29,30,42,100].

Experimental studies have also suggested that S100A8/A9 may contribute to the formation of pre-metastatic niches in distant organs. In this model, inflammatory signals generated by the primary tumor stimulate the production and release of S100A8/A9 from myeloid cells. The resulting accumulation of calprotectin in distant tissues can promote the recruitment of additional immune cells and create a microenvironment that facilitates metastatic colonization [44,101,102,103].

Additional studies suggest that inflammatory chemokine signaling pathways may cooperate with S100-mediated immune regulation to promote metastatic dissemination [104].

The involvement of calprotectin in tumor biology highlights how the unique proteomic architecture of neutrophils can influence disease processes beyond antimicrobial defense. The massive intracellular reservoir of S100A8/A9 allows neutrophils to release large quantities of this protein complex during inflammation, thereby shaping immune responses within the tumor microenvironment [29].

### 7.3. Calprotectin as a Biomarker and Therapeutic Target

Given its strong association with inflammation and tumor progression, S100A8/A9 has attracted considerable interest as a potential biomarker and therapeutic target. Elevated levels of calprotectin have been detected in various biological fluids, including serum, plasma, and feces, and these measurements are increasingly used as indicators of inflammatory activity [20,21,31,43].

In oncology, increased expression of S100A8/A9 has been reported in several cancer types and has been associated with tumor progression, metastasis, and poor clinical outcomes in some contexts [97,99,100].

Experimental evidence also indicates that S100 proteins can influence tumor-associated immune regulation through effects on immune cell recruitment, endothelial integrity, and inflammatory signaling [91,92,105,106,107].

At the same time, the functional roles of S100A8/A9 in inflammatory signaling and immune cell recruitment have prompted investigations into potential therapeutic strategies aimed at targeting calprotectin pathways. Experimental approaches have explored the inhibition of S100A8/A9 signaling or its interaction with receptors such as RAGE and TLR4 in order to modulate inflammatory responses and tumor-associated immune regulation [29,30,31,42,43].

Although these strategies remain under investigation, they highlight the broader biomedical significance of calprotectin biology and the potential clinical relevance of understanding why neutrophils accumulate such unusual quantities of this protein complex.

## 8. Outstanding Questions and Future Directions

Despite decades of research on S100A8 and S100A9, fundamental questions regarding the biological rationale for their remarkable abundance in neutrophils remain unresolved. This conclusion is supported by both early biochemical studies and recent high-throughput proteomic and transcriptomic analyses, indicating that the enrichment of S100A8/A9 is a consistent and robust feature of neutrophil biology rather than an artifact of specific experimental approaches. Although numerous studies have characterized the antimicrobial, inflammatory, and metal-binding properties of calprotectin, these functions alone do not fully explain why neutrophils devote such a large fraction of their cytosolic proteome to this protein complex [20,21,25,29].

One important unresolved issue concerns the intracellular functions of S100A8/A9 within neutrophils. While the extracellular roles of calprotectin as an antimicrobial factor and damage-associated molecular pattern have been extensively investigated, the potential intracellular roles of this protein complex remain less clearly understood [29,30,42]. Given its extremely high cytosolic concentration, it is plausible that S100A8/A9 participates in intracellular processes such as metal buffering, regulation of oxidative stress, or modulation of cytoskeletal dynamics. However, direct experimental evidence supporting these possibilities remains limited [36].

Another key question relates to the developmental regulation of S100A8/A9 expression during granulopoiesis. Although transcription factors such as members of the C/EBP family, PU.1, and GFI1 are known to regulate neutrophil differentiation, the specific regulatory mechanisms responsible for the extraordinarily high expression of S100A8 and S100A9 remain incompletely defined [49,54,58,62]. Understanding how these genes become so strongly expressed during neutrophil maturation may provide important insights into the broader principles governing neutrophil proteome organization.

The evolutionary origins of calprotectin enrichment also remain unclear. The remarkable conservation of S100A8/A9 expression across mammalian species suggests that strong selective pressures have favored this system [21,25,29]. However, whether the primary evolutionary driver of calprotectin abundance relates to antimicrobial defense, regulation of inflammatory signaling, metal homeostasis, or intracellular buffering remains uncertain.

Advances in modern experimental approaches may help clarify these unresolved issues. Quantitative proteomics and single-cell transcriptomic analyses can provide more detailed insights into neutrophil proteome composition and gene expression dynamics during granulopoiesis [22,54,65]. In addition, genetic models targeting S100A8/A9 expression may help reveal how the absence of or reduction in calprotectin affects neutrophil biology and host defense mechanisms [33,34].

Another promising direction involves investigating the role of calprotectin in the broader context of immune system organization. Because neutrophils are among the most abundant immune cells in circulation, the extraordinary abundance of S100A8/A9 may influence systemic immune responses and inflammatory signaling beyond individual cellular functions [3,7,8]. Understanding how calprotectin contributes to immune system dynamics at the tissue and organismal levels may therefore provide new insights into inflammatory disease and tumor-associated immune regulation.

Ultimately, resolving the abundance paradox of S100A8/A9 in neutrophils may reveal fundamental principles governing the organization of innate immune cell proteomes. The predominance of calprotectin within neutrophils represents one of the most striking examples of proteomic specialization in the immune system [21,25]. Elucidating the biological logic underlying this phenomenon may deepen our understanding of how innate immune cells balance efficiency, versatility, and rapid responsiveness during host defense.

## 9. Conclusions

Neutrophils represent one of the most specialized effector cells of the innate immune system, and their proteomic architecture reflects an evolutionary strategy optimized for rapid antimicrobial defense. Unlike many immune cells that depend on inducible transcriptional responses, neutrophils rely on a preassembled repertoire of effector proteins synthesized during granulopoiesis. Within this framework, the remarkable enrichment of S100A8 and S100A9 in the neutrophil cytosol represents one of the most striking examples of proteomic specialization in immune cells [1,3,8,21].

The structural and biochemical properties of the S100A8/A9 complex provide several potential explanations for this unusual abundance. Calprotectin integrates multiple functional capabilities—including antimicrobial metal sequestration, modulation of inflammatory signaling, regulation of oxidative stress, and interactions with intracellular targets—within a single protein complex [25,29,40]. These multifunctional characteristics may enable neutrophils to allocate a substantial fraction of their proteome to a molecule capable of contributing simultaneously to several key aspects of innate immune defense.

At the same time, the extraordinary intracellular accumulation of calprotectin continues to raise important biological questions. Although its antimicrobial and inflammatory activities are well-established, these functions alone may not fully account for the massive proteomic investment observed in neutrophils. The apparent predominance of S100A8/A9 likely reflects a combination of developmental programming, evolutionary selection for multifunctional effector molecules, and the unique physiological constraints under which neutrophils operate [20,25,29].

Understanding the abundance paradox of calprotectin may therefore provide broader insights into how innate immune cells organize their proteomes to balance efficiency, versatility, and rapid responsiveness. Future studies integrating proteomics, genetics, and systems-level analyses of neutrophil biology will be essential for elucidating the precise mechanisms that drive the unusual accumulation of S100A8/A9 and for determining how this unique proteomic configuration contributes to host defense, inflammatory disease, and tumor-associated immune regulation. More broadly, the unusual abundance of calprotectin may reflect a biological strategy in which neutrophils prioritize multifunctional effector proteins capable of simultaneously supporting antimicrobial defense, inflammatory signaling, and cellular stress regulation. Understanding this principle of proteomic prioritization may provide new insights into the evolutionary design of innate immune cells.

## Data Availability

No new data were created or analyzed in this study. Data sharing is not applicable to this article.

## Abbreviations

S100A8	S100 calcium-binding protein A8
S100A9	S100 calcium-binding protein A9
DAMP	damage-associated molecular pattern
TLR4	Toll-like receptor 4
RAGE	receptor for advanced glycation end products
ROS	reactive oxygen species
NETs	neutrophil extracellular traps
NETosis	neutrophil extracellular trap formation
MDSCs	myeloid-derived suppressor cells
MPO	myeloperoxidase
ELANE	neutrophil elastase
NOX2	NADPH oxidase 2
ATP	adenosine triphosphate
C/EBP	CCAAT/enhancer-binding protein
PU.1	ETS family transcription factor PU.1
GFI1	growth factor independent 1
TME	tumor microenvironment
Ca^2+^	calcium ion
Zn^2+^	zinc ion
Mn^2+^	manganese ion
